# Combined effect of CO_2_ enrichment and foliar application of salicylic acid on the production and antioxidant activities of anthocyanin, flavonoids and isoflavonoids from ginger

**DOI:** 10.1186/1472-6882-12-229

**Published:** 2012-11-23

**Authors:** Ali Ghasemzadeh, Hawa ZE Jaafar, Ehsan Karimi, Mohd Hafiz Ibrahim

**Affiliations:** 1 Department of Crop Science, Faculty of Agriculture, University Putra Malaysia, 43400 University Putra Malaysia (UPM), Serdang, Selangor, Malaysia

**Keywords:** CO_2_ enrichment, Salicylic acid, Chalcone synthase, Flavonoids, DPPH activity, Ginger

## Abstract

**Background:**

The increase in atmospheric CO_2_ concentration caused by climate change and agricultural practices is likely to affect biota by producing changes in plant growth, allocation and chemical composition. This study was conducted to evaluate the combined effect of the application of salicylic acid (SA, at two levels: 0 and 10^-3^ M) and CO_2_ enrichment (at two levels: 400 and 800 μmol·mol^−1^) on the production and antioxidant activities of anthocyanin, flavonoids and isoflavonoids from two Malaysian ginger varieties, namely Halia Bentong and Halia Bara.

**Methods:**

High-performance liquid chromatography (HPLC) with photodiode array detection and mass spectrometry was employed to identify and quantify the flavonoids and anthocyanins in the ginger extracts. The antioxidant activity of the leaf extracts was determined by the 1,1-diphenyl-2-picrylhydrazyl (DPPH) and thiobarbituric acid (TBA) assays. The substrate specificity of chalcone synthase, the key enzyme for flavonoid biosynthesis, was investigated using the chalcone synthase (CHS) assay.

**Results:**

CO_2_ levels of 800 μmol·mol^−1^ significantly increased anthocyanin, rutin, naringenin, myricetin, apigenin, fisetin and morin contents in ginger leaves. Meanwhile, the combined effect of SA and CO_2_ enrichment enhanced anthocyanin and flavonoid production compared with single treatment effects. High anthocyanin content was observed in H Bara leaves treated with elevated CO_2_ and SA. The highest chalcone synthase (CHS) activity was observed in plants treated with SA and CO_2_ enrichment. Plants not treated with SA and kept under ambient CO_2_ conditions showed the lowest CHS activity. The highest free radical scavenging activity corresponded to H Bara treated with SA under high CO_2_ conditions, while the lowest activity corresponded to H Bentong without SA treatment and under atmospheric CO_2_ levels. As the level of CO_2_ increased, the DPPH activity increased. Higher TBA activity was also recorded in the extracts of H Bara treated with SA and grown under high CO_2_ conditions.

**Conclusions:**

The biological activities of both ginger varieties were enhanced when the plants were treated with SA and grown under elevated CO_2_ concentration. The increase in the production of anthocyanin and flavonoids in plants treated with SA could be attributed to the increase in CHS activity under high CO_2_ levels.

## Background

**Figure 1:**
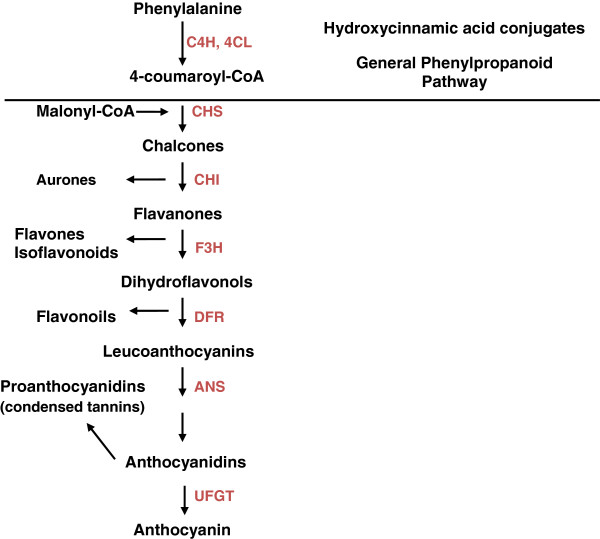
**Schematic representation of the flavonoid biosynthetic pathway.** Enzyme abbreviations: PAL, phenylalanine ammonia-lyase; C4H, cinnamate 4-hydroxylase; 4CL, 4-coumaroyl:CoA ligase; CHS, chalcone synthase; CHI, chalcone isomerase; F3H, flavanone 3-hydroxylase; DFR, dihydroflavonol 4-reductase; ANS, anthocyanidin synthase; UFGT, UDP glucose-flavonoid 3-*O*-glucosyl transferase [10].

Phytochemicals and antioxidants from plant sources are of increasing interest to consumers because of their roles in the maintenance of human health. Plants are a rich source of various phytochemicals, proteins, enzymes and other products of immense biotechnological value. Most of the secondary metabolites of herbs and spices are commercially important and are used in a number of pharmaceutical products. Flavonoids are the most important group of secondary metabolites and bioactive compounds in plants [1]. Flavonoids are known for their health-promoting properties, which include protective effects against cardiovascular disease, cancer and other diseases. They also have antioxidant properties, being capable of scavenging free superoxide radicals, as well as having anti-aging and anticancer activities [2]. It was found that flavonoids reduce blood lipid and glucose, and enhance human immunity [3]. The effect of flavonoids on human health is the result of their ability to induce human protective enzyme systems [4]. Several studies have suggested that flavonoids such as catechin and quercetin are able to control cancer cell growth in the human body [5-7]. The flavonoid biosynthetic pathway starts with the condensation of one molecule of 4-coumaroyl-CoA and three molecules of malonyl-CoA, yielding naringenin chalcone. This reaction is carried out by chalcone synthase (CHS). Chalcone is isomerised to a flavanone by the enzyme chalcone flavanone isomerase (CHI). From these central intermediates, the pathway diverges into several side branches, each resulting in a different class of flavonoids. Flavanone 3-hydroxylase (F3H) catalyses the stereospecific 3ß-hydroxylation of (2S)-flavanones to dihydroflavonols. In the biosynthesis of anthocyanins, dihydroflavonol reductase (DFR) catalyses the reduction of dihydroflavonols to flavan-3,4-diols (leucoanthocyanins), which are converted to anthocyanidins by anthocyanidin synthase (ANS). The formation of glucosides is catalysed by UDP glucose-flavonoid 3-*O*-glucosyl transferase (UFGT), which stabilises the anthocyanidins by 3-*O*-glucosylation [8,9]. The overview of the flavonoid pathway is presented in Figure 1. There is evidence that the enzymes involved in the flavonoid metabolism act as membrane-associated multi-enzyme complexes, which has implications on the overall efficiency, specificity, and regulation of the pathway [10]. Anthocyanin is the water-soluble pigment which imparts the red, purple, and blue coloration to many fruits, vegetables, and cereal grains. This pigment is largely responsible for the colour characteristics of raw and processed products. Anthocyanin is frequently used as a food additive and it has been recognised that procyanidin has anti-carcinogenic and anti-oxidant activities [11].

Salicylic acid (SA) is a phenolic compound capable of enhancing plant growth and yield in some plants [12]. SA acts as a potential non-enzymatic antioxidant, as well as a plant growth regulator, and plays an important role in regulating a number of plant physiological processes, including photosynthesis [13,14]. Previous reports showed that exogenous SA could ameliorate the damaging effects of heavy metals in rice [15], drought stress in wheat [14], and salt stress in wheat [12]. These observations suggested that SA could be linked to oxidative stress. Furthermore, other reports indicated that CO_2_ enrichment increased the production of secondary metabolites [16,17] and the antioxidant activity of plants [18]. Enrichment with high CO_2_ levels has been shown to enhance the medicinal properties of some plants, including *Labisia pumila* Blume (known in Malaysia as Kacip Fatimah) [19], oil palm [20], ginger [21], and strawberry [17]. According to the carbon-nutrient balance theory, as the carbon to nitrogen ratio increases under an elevated atmospheric CO_2_ environment, a greater amount of the plant’s carbohydrates can be allocated to the plant’s secondary metabolism, resulting in the production of greater amounts of carbon-based secondary metabolites [22]. Ginger (*Zingiber officinale* Roscoe) is a famous and widely used herb, especially in Asia, which contains several interesting bioactive constituents possessing health-promoting properties. In Malaysia, ginger has been used as a food and medicinal plant for over 2000 years to treat diabetes, high blood pressure, cancer and many other illnesses [23]. However, there has been little discussion about the combined effect of SA and CO_2_ enrichment on the production of secondary metabolites generated by the phenylpropanoid pathway and their antioxidant activity in plants.

Hence, the aim of the present study was to examine the combined effect of SA and CO_2_ enrichment on the production and antioxidant activity of anthocyanin, flavonoids, and isoflavonoids from two Malaysian ginger varieties. We also measured the CHS activity with salicylic acid stimulation.

## Methods

### Plant materials

Rhizomes from two ginger varieties (Halia Bentong and Halia Bara) were collected from a village in Bentong, Pahang, Malaysia. Rhizomes were soaked in a Mancozeb solution for 30 min to give a final concentration of 0.3%. Pots filled with about 1 kg peat moss were prepared. Rhizomes were cut into 3–5 cm pieces containing 2 to 3 buds, and were planted 6 cm deep into the peat moss, with the buds facing upward. Rhizomes were grown in a glasshouse for two weeks. Afterward, when the young leaves reached a height of 5 cm, seedlings were transplanted into polyethylene bags filled with a soilless mixture composed of burnt rice husk and coco peat (1:1).

### Arrangement of carbon dioxide enrichment system

Ginger seedlings were transferred to a CO_2_ growth chamber (Conviron EF7, Canada) with two different CO_2_ concentrations: 400 μmol·mol^-1^ as ambient and 800 μmol·mol^-1^ as elevated CO_2_ concentration. Pure carbon dioxide (99.8% purity; Science Gates Sdn Bhd) was supplied from a high concentration carbon dioxide cylinder (22.67 kg, pressure 2200 psi) and injected through a pressure regulator into the closed fumigation chamber. The pressure was set to no more than 5 bar for safety reasons. The flow and concentration of carbon dioxide in the chamber was monitored and controlled with a CO_2_ PPM3 Controller™. Enrichment was done automatically according to the treatment. Rotating fans (left and right side) were used to disperse the carbon dioxide evenly inside the chamber. Photoperiod (310 μmol·m^-2^ s^-1^), relative humidity (70–80%) and air temperature (25–33°C) were controlled using an integrated control, monitoring, and data management system (Dynamac Corp., Rockville, MD, USA.). Carbon dioxide was supplied for 2 h daily, applied continuous from 08:00 to 10:00 a.m. [24]. When the ginger seedlings were at the second leaf stage, they were sprayed with 10^–3^ M salicylic acid (SA) solution (2-hydroxybenzoic acid + 100 μL dimethyl sulfoxide + 0.02% polyoxyethylene sorbitan monolaurate, Tween 20; Sigma Chemicals, pH 6.5). Control plants were sprayed with the same solution but without SA. Leaves were sprayed once early in the morning and every week until the 4th month. A nutrient solution in a fertigation system (recommended mixture fertilizer for ginger) was also applied. This factorial experiment was arranged in a split plot using a randomized complete block design replicated three times. Each treatment consisted of ten seedlings.

### Extraction of anthocyanin and flavonoids

Approximately 1 g of plant powder was extracted with 5 mL of methanol containing 0.1% HCl (pH 2.8) at 4°C for 24 h in a dark room, vortexed every 6 h. The liquid was separated from the solid matrix by filtration through sheets of qualitative filter paper (Hangzhou Special Paper Industry, Zhejiang, China). The filtrate was further passed through 0.22 μm reinforced nylon membrane filters (Shanghai ANPEL, Shanghai, China) before HPLC analysis. Three replicates were performed for each sample.

### HPLC analysis of anthocyanin and flavonoids

Reversed-phase HPLC was used to assay flavonoid composition. The HPLC system (Agilent Technologies Inc., Palo Alto, CA) consisted of a Model 1100 pump equipped with a multi-solvent delivery system and an L-7400 ultraviolet (UV) detector. The analytical column was an ODS-80Ts QA C18 column (150 mm × 4.6 mm I.D.; Tosoh, Tokyo, Japan) protected with a Transgenomic CARB Sep Coregel 87C Guard Cartridge (Transgenomic, Omaha, NE, USA). Absorbance spectra were collected for all peaks. The solvent flow rate was 1.0 ml·min^-1^. The injection volume was 25 μL. Solvent A was 100% acetonitrile and solvent B consisted of 10% (v/v) acetic acid and 1% (v/v) phosphoric acid in water. Chromatograms were acquired at 520 and 280–365 nm for anthocyanin and flavonoids, respectively. The system was operated at room temperature. Quantification was performed by the external standard method [25].

### Antioxidant activities

#### 1,1-Diphenyl-2-picrylhydrazyl (DPPH) assay

Free radical scavenging activity was determined according to the method described by Mensor et al. [26]. A DPPH alcohol solution (3 mL) was added to 1 mL samples containing different concentrations of extracts originating from different ginger parts. Samples were first kept in a dark place at room temperature and their absorbance was read at 518 nm after 30 min. The antiradical activity was determined using the formula below:

$$\text{Percent}\;\left(\%\right)\;\text{inhibition of DPPH activity}=\left[\left({\text{A}}_{0}–{\text{A}}_{1}\right)/{\text{A}}_{0}\right)]\times 100\%$$

Where A_0_ is the absorbance value of the blank sample or control reaction, and A_1_ is the absorbance value of the test sample. The optic densities of the samples, controls and blanks were measured in comparison with ethanol. BHT (butylhydroxytoluene) and α-tocopherol were used as positive controls. The parameter IC_50_ was calculated graphically. A lower IC_50_ value indicates greater antioxidant activity.

#### Thiobarbituric acid (TBA) assay

Various concentrations of test samples (10–500 μg·mL^-1^) were added to an aqueous solution (2 mL) containing 200 μL of Tris buffer (pH 7.4), 300 μL of 1 M KCl, 400 μL of 1% SDS (sodium dodecyl sulfate), 10 μL of linolenic acid, 40 μL of 1.0 μM FeCl_2_, and 20 μL of 0.5 μM H_2_O_2_ in a brown non-transparent vial (to avoid any oxidation caused by UV irradiation). The sample vial was then incubated for 18 h at 37°C while being shaken. After incubation, oxidation was terminated by adding 50 μL of 4% solution of BHT in ethanol, followed by the addition of 2 mL of TBA reagent (0.67% TBA). The mixture was heated at 80°C for 1 h and then cooled down in an ice bath for 10 min. A blank was prepared following the same procedure but without a test sample. The TBA-MA adduct formed was measured using a spectrophotometer at 532 nm. Known antioxidants (BHT and α-tocopherol) were used as positive controls [27].

### Chalcone synthase (CHS) activity

CHS activity was assayed spectrophotometrically, as described by Obinata et al. [28]. Enzyme was extracted at 4°C by homogenising the harvested frozen cells (0.4 g) in 1 mL of 0.1 M borate buffer (pH 8.8) containing 1 mM 2-mercaptoethanol. The homogenates were treated with 0.1 g of Dowex l×4 for 10 min and the cell debris and resin were removed by centrifugation at 15000 rpm for 10 min. Dowex l×4 resin (0.2 g) was added to the supernatant and treated for another 20 min. Then, the resin was removed by centrifugation at 15000 rpm for 15 min. The resultant supernatant was used for the CHS assays. For this, 100 μL of enzyme extract was mixed with 1.89 mL of 50 mM Tris-HCI buffer, pH 7.6, containing 10 mM KCN. The enzyme reaction was allowed to proceed for 1 min at 30°C after adding 10 mg of chalcone in 10 μL ethylene glycol monomethyl ether. The activity was determined by measuring the absorbance at 370 nm.

### Statistical analysis

All analytical values shown represent the means of three replicates. Data were analysed using analysis of variance by Statistical Analysis System (SAS 9.0). Mean separation test between treatments was performed using Duncan multiple range test and a *P*-value ≤ 0.05 was regarded as significant.

## Results and discussion

### HPLC analysis of anthocyanin, flavonoids and isoflavonoids

Carbon dioxide levels had a significant (*P* ≤ 0.01) impact on the production of anthocyanin and other flavonoids in both ginger varieties (Table 1). As CO_2_ levels increased from 400 to 800 μmol·mol^−1^, flavonoid production was enhanced. High CO_2_ conditions significantly enhanced the anthocyanin, rutin, naringenin, myricetin, apigenin, fisetin, and morin contents in ginger leaves. Leaves from plants grown under ambient CO_2_ conditions had the lowest content of these flavonoids. Plants treated with SA produced higher concentrations of anthocyanin and flavonoids compared with plants kept under high CO_2_ concentration but without SA treatment. The combined effect of SA and CO_2_ enrichment resulted in significant enhancement of anthocyanin and flavonoid production compared with the single individual treatments. High anthocyanin content was observed in H Bara leaves (0.355 mg·g^-1^ DW) treated with elevated CO_2_ and 10^-3^ M SA. The lowest content of anthocyanin was observed in H Bentong (0.245 mg·g^-1^ DW) grown under ambient CO_2_ with no SA treatment.

**Table 1:** **The concentrations of anthocyanin and some flavonoid compounds in two varieties of ginger, treated with SA and grown under different CO**_**2**_**concentrations (400 and 800** μmol·mol ^**-1**^**CO**_**2**_**)**

**Variety**	**CO**_**2**_**(μmol·mol**^**-1**^**)**	**SA (M)**	**Anthocyanin**	**Rutin**	**Naringenin**	**Myricetin**	**Apigenin**	**Fisetin**	**Morin**
H.Bentong	400	0	0.245±0.007 ^g^	0.68±0.023 ^c^	0.058±0.0014 ^de^	0.119±0.0016 ^e^	0.32±0.0148 ^d^	0.78±0.017 ^h^	0.545±0.007 ^e^
		10^-3^	0.276±0.0051 ^ef^	0.766±0.0077 ^c^	0.105±0.0035 ^c^	0.404±0.0021 ^a^	0.204±0.01 ^e^	1.13±0.0056 ^g^	0.597±0.014 ^d^
	800	0	0.297±0.0027 ^de^	0.924±0.0098 ^b^	0.093±0.0023 ^cd^	0.184±0.0014 ^d^	0.593±0.024 ^ab^	2.16±0.144 ^d^	0.503±0.027 ^f^
		10^-3^	0.306±0.0084 ^cd^	0.933±0.0374 ^b^	0.166_0.017 ^a^	0.344±0.0134 ^b^	0.552±0.0183 ^b^	2.93±0.101 ^b^	0.541±0.019 ^e^
H.Bara	400	0	0.273±0.0035 ^f^	0.773±0.015 ^c^	0.039±0.0013 ^f^	0.127±0.0033 ^e^	0.571±0.041 ^b^	1.44±0.063 ^f^	0.686±0.0035 ^b^
		10^-3^	0.334±0.0146 ^ab^	1.003±0.0622 ^ab^	0.072±0.0021 ^de^	0.288±0.0035 ^c^	0.44±0.018 ^c^	1.69±0.064 ^e^	0.849±0.012 ^a^
	800	0	0.325±0.0155 ^bc^	1.04±0.0551 ^a^	0.133±0.0056 ^b^	0.201±0.013 ^d^	0.644±0.0077 ^a^	2.47±0.21 ^c^	0.592±0.013 ^d^
		10^-3^	0.355±0.0148 ^a^	1.096±0.07 ^a^	0.148±0.014 ^ab^	0.355±0.0084 ^b^	0.598±0.0261 ^ab^	3.23±0.098 ^a^	0.618±0.015 ^c^

All analyses are the mean of triplicate measurements ± standard deviation; Results expressed in mg g^-1^DW; Means not sharing a common letter were significantly different at P ≤ 0.05.

An interesting finding was that plants treated with SA exhibited a lower content of apigenin compared with untreated plants. According to the results in Table 1, both ginger varieties treated with 10^-3^ M SA and kept under ambient and elevated CO_2_ conditions had significantly lower concentrations of apigenin. A high content of apigenin (0.644 mg·g^-1^ DW) was detected in H Bara without SA treatment and kept at 800 μmol·mol^-1^ CO_2_, while a low content (0.204 mg·g^-1^ DW) of this isoflavonoid was detected in H Bentong treated with 10^-3^ M SA and kept under ambient CO_2_ conditions (400 μmol·mol^-1^CO_2_). A similar trend of increasing concentrations of flavonoids with increasing CO_2_ concentration was observed in *Scutellaria* species [29]. Wang et al. [17] reported that strawberry plants under CO_2_ enrichment conditions (950 μmol·mol^-1^CO_2_) had a significantly increased *p*-coumaroylglucose, dihydroflavonol, quercetin 3-glucoside, quercetin 3-glucuronide, and kaempferol 3-glucoside contents in fruit, as well as increased cyanidin 3-glucoside, pelargonidin 3-glucoside, and pelargonidin 3-glucoside succinate contents.

**Figure 2:**
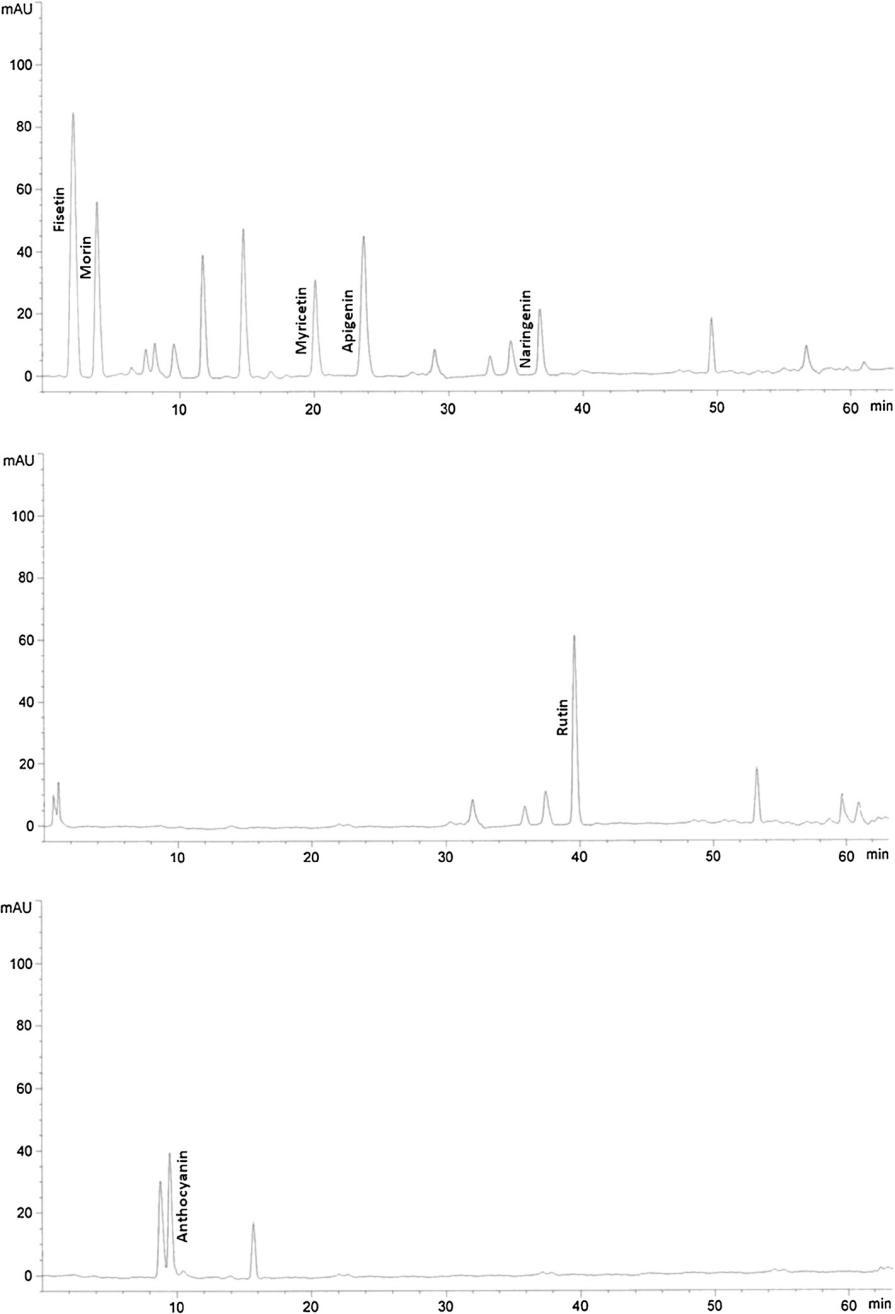
**HPLC chromatogram of leaf extracts of H. Bara ginger treated with 10**^**-3**^**M SA and kept under elevated CO**_**2**_**condition (800 μmol·mol**^**-1**^**CO**_**2**_**).**

Fisetin is a rare yet well-known flavonoid compound in plants. Previous studies have shown that fisetin has anti-inflammatory [30,31], anti-carcinogenic [32] and strong antioxidant effects. Ginger leaves contained relatively high levels of this flavonoid. It was apparent that fisetin content could also be improved by increasing CO_2_ concentration coupled with SA treatment in both ginger varieties, and especially in H Bara (2.47 mg·g^-1^ DW increased to 3.23 mg·g^-1^ DW). Morin also belongs to the flavonol group, and acts as a chemopreventive agent *in vitro* and *in vivo* against oral carcinogenesis [33]. The importance of morin and related compounds as anti-tumour drugs has been widely recognised [34]. In comparison with old fustic (*Chlorophora tinctoria*), osage orange (*Maclura pomifera*), and almond (*Prunus dulcis*) [35], the local ginger varieties showed good levels of morin when grown under 800 μmol·mol^-1^ CO_2_ levels coupled with 10^-3^ M SA treatment, indicating that the plant is naturally a good source of morin, although the content was variable. The content of morin in leaves decreased in both varieties with increasing CO_2_ concentration. However, a high content of morin was detected in H Bara (0.849 mg·g^-1^ DW) treated with SA under ambient CO_2_ condition. Thus, in ginger treated with SA under ambient CO_2_ conditions, the concentration of morin in leaves was enhanced, while under elevated CO_2_ levels, the concentration of this flavonoid decreased in both varieties (Table 1). Figure 2 shows the HPLC chromatogram of H Bara leaf extracts from plants treated with 10^-3^ M SA under elevated CO_2_ condition (800 μmol·mol^-1^ CO_2_).

### Chalcone synthase (CHS) activity

**Figure 3:**
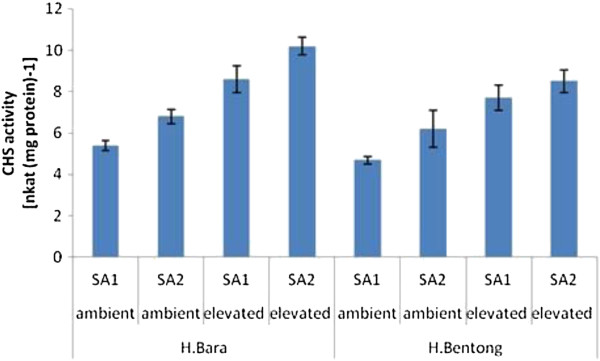
**CHS activity in two ginger varieties treated with SA and CO**_**2**_**enrichment (SA1=non SA, SA2=10**^**-3**^**M SA, ambient=400 μmol·mol**^**−1**^**CO**_**2**_**, elevated=800 μmol·mol**^**−1**^**CO**_**2**_**).** Error bars represent the standard error of means.

CHS activity was influenced by SA and CO_2_ concentrations (*P* ≤ 0.01; Figure 3). In both ginger varieties treated with SA, the highest CHS activity was consistently found in plants kept under 800 μmol·mol^−1^ CO_2_, with values ranging between 8.5 and 10.2 nkat·mg protein^-1^. In contrast, plants subjected to the 400 μmol·mol^−1^ CO_2_ treatment had a CHS activity between 6.2 and 6.8 nkat·mg protein^-1^. Plants kept under ambient CO_2_ conditions and not treated with SA showed the lowest CHS activity, with values between 4.7 and 5.4 nkat·mg protein^-1^. The present study shows that CHS activity was enhanced with an increase in CO_2_ levels coupled with the application of SA. This is mainly because CHS is a precursor to flavonoid biosynthesis [36,37]. The increase in CHS activity is usually followed by an increase in the C/N ratio derived from the enhanced growth rate under elevated CO_2_. Recent studies have indicated that an increase in the C/N ratio in plants corresponded to an increase in the synthesis of secondary metabolites, especially flavonoids [20,38]. An increase in PAL activity (another enzyme involved in flavonoid synthesis) under high CO_2_ has also been observed in tobacco and *Spergula avensis*[39-41].

Thus, the increase in the production of anthocyanin and flavonoids reported in the present work could be attributed to an increase in CHS activity under high CO_2_ levels. Ozeki et al. [42] pointed out that changes in CHS activity, rather than PAL activity, were correlated with changes in anthocyanin accumulation under various culture conditions. CHS is the first enzyme to branch off from phenylpropanoid metabolism to flavonoid metabolism and is believed to be a key enzyme of this system [10]. These findings, together with evidence for channelling between CO_2_ enrichment and CHS activity in the general phenylpropanoid pathway, indicate that the organisation of these systems is important for understanding how plant metabolism is regulated.

### Antioxidant activity

#### 1,1-Diphenyl-2-picrylhydrazyl (DPPH) assay

The DPPH stable free radical method is an easy, rapid and sensitive way to evaluate the antioxidants that scavenge free radicals. As shown in Table 2, DPPH activities in ginger leaf were influenced by CO_2_ levels and SA treatment (*P* ≤ 0.01). Generally, the highest DPPH activity was recorded in H Bara (70.27%) treated with SA and kept under 800 μmol·mol^−1^ CO_2_ and the lowest activity was observed in H Bentong (38.29%) not treated with SA and kept under 400 μmol·mol^−1^ CO_2_. As the levels of CO_2_ increased, the DPPH activities were enhanced. DPPH activities corresponding to elevated CO_2_ treatment (800 μmol·mol^−1^ CO_2_) were in the range 61.88% to 70.22%. In contrast, DPPH activities corresponding to 400 μmol·mol^−1^ CO_2_ treatment ranged from 38.29% to 45.35%, suggesting that elevated CO_2_ conditions were able to enhance the antioxidant properties of the ginger leaves. It was interesting that the antioxidant activity in CO_2_-treated plants increased significantly when 10^-3^ M SA was applied. The increase in the DPPH activity of H Bentong and H Bara might be due to the high anthocyanin and flavonoid content in these varieties. Significant positive correlations between DPPH activity and flavonoids (*P* ≤ 0.01) were observed, implying that the enhanced antioxidant activity detected in plants kept under high levels of CO_2_ coupled with SA treatment could be related to the increased hydrogen donating abilities of the plants [43,44].

**Table 2:** **DPPH scavenging activities and IC**_**50**_**values of the methanolic extracts of two varieties of *Zingiber officinale *treated with SA and grown under different CO**_**2**_**concentrations (ambient: 400 μmol·mol**^**−1**^**CO**_**2**_**and elevated: 800 μmol·mol**^**−1**^**CO**_**2**_**)**

**Variety**	**CO2(μmol·mol**^**−1**^**)**	**SA (M)**	**DPPH (%)**	**IC**_**50**_**(μg g**^**-1**^**)**
H.Bentong	400	0	38.29±0.55 ^g^	37.4
		10^-3^	52.98±1.209 ^e^	35.8
	800	0	61.88±0.636 ^d^	34.2
		10^-3^	68.075±0.403 ^ab^	30.55
H.Bara	400	0	45.35±0.82 ^f^	28.2
		10^-3^	63.7±2.52 ^cd^	25.6
	800	0	65.91±0.19 ^bc^	23.71
		10^-3^	70.27±0.431 ^a^	21.46
Positive controls	α-tocopherol		93.47±0.77	16.2
	BHT		89.27±1.04	20.4

All analyses are the mean of triplicate measurements ± standard deviation; Means not sharing a common letter were significantly different at P < 0.05.

For both ambient and elevated CO_2_ conditions, ginger leaves treated with 10^-3^ M SA exhibited higher radical scavenging activity than leaves from untreated plants (Table 2). For H Bara kept under 400 μmol·mol^−1^ CO_2_ treatment, the antioxidant activity was enhanced by about 28.8% for plants treated with SA. The IC_50_ (fifty percent free radical scavenging) value changed significantly after SA treatment. In both varieties, the IC_50_ in SA-treated gingers was observed to be lower compared with untreated ginger. Also, the IC_50_ was observed to be lower in CO_2_-enriched plants. The H Bara variety treated with SA and grown under elevated CO_2_ concentration (800 μmol·mol^−1^ CO_2_) exhibited a lower IC_50_ value (21.46 μg·ml^-1^).

The DPPH values of leaf extracts of both ginger varieties treated with SA and grown under two different CO_2_ concentrations (400 and 800 μmol·mol^−1^ CO_2_) were significantly lower than those of α-tocopherol (93.47%) and BHT (89.27%) (Table 2). It was evident that CO_2_ enrichment significantly enhanced flavonoid content in both ginger varieties, and the high flavonoid content was associated with high antioxidant activity.

Our results agree with the findings of previous studies. A positive relationship between phenolics and flavonoids with free radical scavenging has been reported in previous studies [45-48]. Our results showed that DPPH activity had a significant positive correlation with most flavonoids (*P* ≤ 0.01 and *P* ≤ 0.05; Table 3).

**Table 3:** Correlation between measured parameters in two ginger varieties

	**Anthocyanin**	**Fisetin**	**Morien**	**Myricetin**	**Apigenin**	**Rutin**	**Naringenin**	**CHS**	**DPPH**	**TBA**
Anthocyanin	1									
Fisetin	0.785^**^	1								
Morien	0.473^ns^	0.505^*^	1							
Myricetin	0.334^ns^	0.248^ns^	0.56^*^	1						
Apigenin	0.577^*^	0.761^**^	0.141^ns^	0.201^ns^	1					
Rutin	0.978^**^	0.833^**^	0.394^ns^	0.19^ns^	0.665^**^	1				
Naringenin	0.58^*^	0.809^**^	0.402^ns^	0.495^ns^	0.446^ns^	0.628^**^	1			
CHS	0.55^*^	0.587^*^	0.447^ns^	0.304^ns^	0.134^ns^	0.368^ns^	0.517^*^	1		
DPPH	0.881^**^	0.886^**^	0.525^*^	0.377^ns^	0.636^**^	0.901^**^	0.793^**^	0.441^ns^	1	
TBA	0.642^**^	0.714^**^	0.462^ns^	0.235^ns^	0.635^**^	0.465^ns^	0.404^ns^	0.276^ns^	0.401^ns^	1

n.s = non significant; * = significant at p ≤ 0.05; ** = significant at p ≤ 0.01.

#### Thiobarbituric acid (TBA) assay

**Figure 4:**
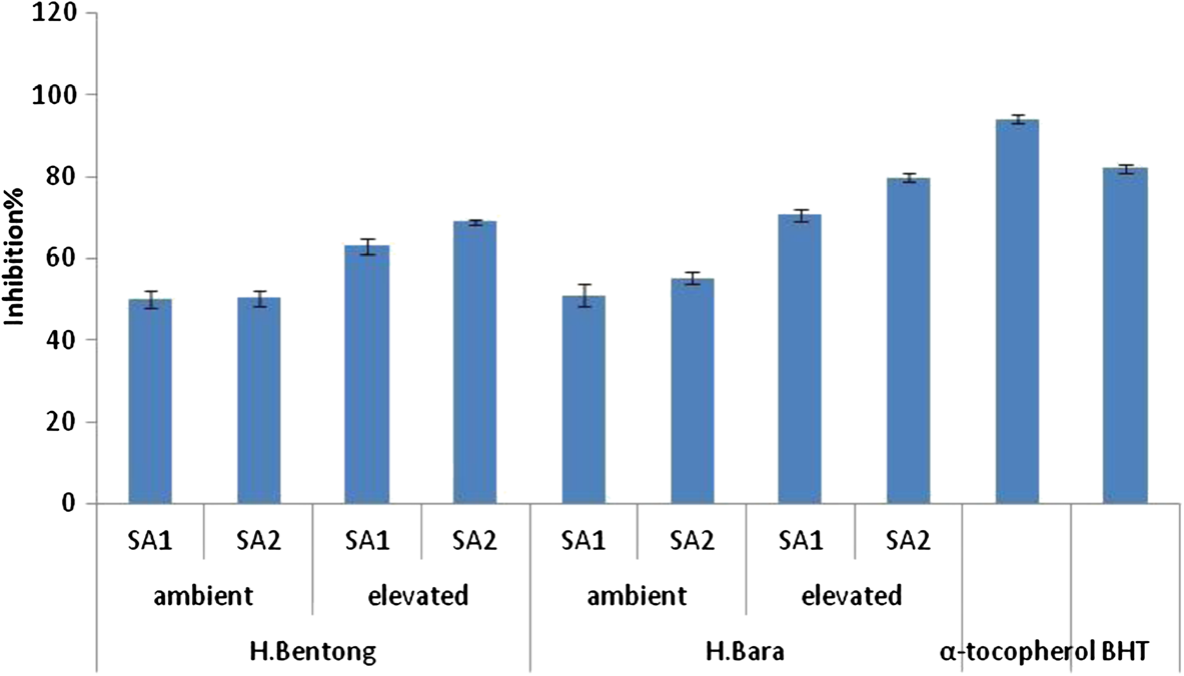
**TBA activity of ginger varieties treated with SA and CO**_**2**_**enrichment (SA**_**1**_**=non SA, SA**_**2**_**=10**^**-3**^**M SA, ambient=400 μmol·mol**^**−1**^**CO**_**2**_**, elevated=800 μmol·mol**^**−1**^**CO**_**2**_**).** Error bars represent standard error of means.

In comparison to control plants, the extracts analysed showed strong antioxidant activity when treated with SA and CO_2_ enrichment (Figure 4). Higher TBA activity was recorded in extracts of H Bara (79.84%) treated with SA (10^-3^ M) and grown under elevated CO_2_ conditions (800 μmol·mol^−1^ CO_2_). The leaf extracts of H Bara and H Bentong treated with SA and exposed to elevated CO_2_ conditions were observed to have medium antioxidant activity (79.84 and 68.96%, respectively) compared with the positive controls α-tocopherol (94.2%) and BHT (82.7%), while plants with no SA treatment and grown under ambient CO_2_ conditions showed the lowest antioxidant activity (51.04 and 50.1%, respectively). Under CO_2_-enriched conditions (800 μmol·mol^−1^ CO_2_) the TBA activity of H Bara (70.5%) with no SA treatment was not significantly different from that of H Bentong (68.96%) treated with SA. Therefore, these results need to be interpreted with caution.

Wang et al. [17] reported that the free radical scavenging power of strawberry increased at elevated CO_2_ concentrations (950 μmol·mol^-1^ CO_2_). Similarly, a high CO_2_ content may have enhanced the antioxidant activity of the ginger extracts. Correlation analyses showed that the increase in antioxidant activity might be up-regulated by an increase in the flavonoid and anthocyanin content of plants treated with SA under elevated CO_2_ (Table 3). Furthermore, CHS showed a positive and significant correlation (*P* ≤ 0.05) with anthocyanin, fisetin, morin and naringenin, although no significant correlation was observed between this enzyme and myricetin, apigenin and rutin. This study has shown that ginger has a remarkable free radical scavenging ability, and therefore can be used as a radical inhibitor or scavenger, acting possibly as a primary antioxidant.

## Conclusion

The results of this study suggest that rising atmospheric concentrations of carbon dioxide could have a major impact on the antioxidant capacity of ginger. Anthocyanin and flavonoid compounds are largely responsible for the antioxidant activity in plant tissues [25]. Anthocyanin is known to reduce the damage caused by free-radical activity, including low-density lipoprotein oxidation, platelet aggregation, and endothelium-dependent vasodilation of arteries. The anthocyanin and flavonoid contents of ginger treated with SA and grown under 800 μmol·mol^-1^ enrichment conditions were significantly higher than those of untreated plants grown under 800 μmol·mol^-1^ CO_2_ or treated with SA and grown under 400 μmol·mol^-1^ CO_2_. Increases in the C/N ratio of plants were an indication of increases in the synthesis of secondary metabolites, especially phenolics and flavonoids [49]. Our results also indicated that CHS activity in ginger can be enhanced by CO_2_ enrichment in a controlled environment (CE). Thus, the production of anthocyanin, flavonoids and isoflavonoids in young ginger could also be increased. The increase in anthocyanin and flavonoids in elevated CO_2_-treated gingers is associated with increased antioxidant capacity in both ginger varieties. The two ginger varieties contain flavonoids with potent antioxidant properties, and under CO_2_ enrichment conditions, both the anthocyanin concentration and the CHS activity increased. The impact of cultural conditions and CO_2_ concentration on biopharmaceutical production in herbs will enable the optimisation of herb chemistry. The composition of flavonoids will be an important consideration in the development of any CE production system for medicinal plants. The present findings have important implications for the development of ginger plantations in Malaysia.

## Competing interests

The authors declare that they have no competing interests.

## Authors’ contributions

Study design and experimental work was by A Ghasemzadeh under the supervision of H Jaafar. The first draft of the paper was written by A Ghasemzadeh and reviewed by H Jaafar. E Karimi and M Ibrahim participated in extraction. All authors reviewed and approved the final version.

## Pre-publication history

The pre-publication history for this paper can be accessed here:

http://www.biomedcentral.com/1472-6882/12/229/prepub
